# Next-Generation Food Packaging: Progress and Challenges of Biopolymer-Based Materials

**DOI:** 10.3390/polym17172299

**Published:** 2025-08-25

**Authors:** Raja Venkatesan, Maher M. Alrashed, Alexandre A. Vetcher, Seong-Cheol Kim

**Affiliations:** 1 School of Chemical Engineering, Yeungnam University, 280 Daehak-Ro, Gyeongsan 38541, Republic of Korea; 2 Department of Biomaterials, Saveetha Dental College and Hospitals, SIMATS, Saveetha University, Chennai 600077, India; 3 Chemical Engineering Department, College of Engineering, King Saud University, P.O. Box 800, Riyadh 11421, Saudi Arabia; 4 Institute of Pharmacy and Biotechnology (IPhB), RUDN University n.a. P. Lumumba (RUDN), 6 Miklukho-Maklaya Str, Moscow 117198, Russia; avetcher@gmail.com

**Keywords:** biopolymers, food packaging, challenges, machinability, migration kinetics, toxicity

## Abstract

Biopolymer-based packaging is emerging as a sustainable replacement for conventional plastic materials. Significant challenges like scalability, machinability, and mechanical properties are preventing biopolymers from industrially advancing despite their sustainable advantages. Also, the usage of materials in packaging is limited due to their toxicity, the degradation products, and their migration properties. Nanocomposite materials and active packaging methods with the antimicrobial agents showed novel advances with enhanced performance. However, these advances frequently increase the complexity and cost of production. For an assessment of their safe and efficient usage, knowledge gaps on the effects of biopolymer migration and degradation on the environment and human health should be addressed. These challenges, which involve enhanced material characteristics, reducing costs, and aligning regulations, demand interdisciplinary methods. This review explores the prospects for sustainable innovation in packaging by examining the challenges and potential solutions associated with the development of biopolymer-based materials.

## 1. Introduction

As an alternative to conventional synthetic polymer packaging, biopolymer-based packaging refers to sustainable and biodegradable packaging materials derived from a natural process, renewable resources [1,2]. In particular, in food packaging, these biodegradable packaging materials have a chance to address major environmental issues associated with plastics. Biopolymers are used to preserve food goods, enhancing their shelf life and safety via active packaging methods in response to the growing need for sustainable practices. A variety of materials, such as moisture absorbers, antioxidants, and antimicrobials, can be used in active packaging to preserve food and enhance its shelf life [3,4]. However, a number of challenges stand in the way of extending the use of biopolymer-based packaging in the food industry. The challenges have included high production costs, poor compatibility with industrial manufacturing methods, low biodegradability, and potential toxicity [5]. All of those issues have an effect on the potential shift away from conventional plastics and towards sustainable alternatives.

It is crucial to evaluate the environmental effect and suitability of biopolymers for food applications by evaluating their degradation behavior in both natural and synthetic environments in in vitro digestion [6]. Product developers can develop safe and effective degradation products by understanding that their components can break down in soil, the human gastrointestinal tract, and marine environments. The effects of degradation products produced during the decomposition of bioplastics on ecosystems and any potential adverse impacts on humans must also be understood. To develop consumer-friendly and sustainable biopolymer-based packaging systems, these processes must be thoroughly understood. Additionally, the potential toxicity of biopolymers and their decomposition products remains a significant concern [7]. The environment and consumers can be at risk due to the potential for some biopolymers or optimization-related additives to produce hazardous materials over time. Based on the direct and prolonged human exposure to food-contact materials, these problems require rigorous tests and regulatory oversight to confirm that these materials meet safety standards.

**Figure 1:**
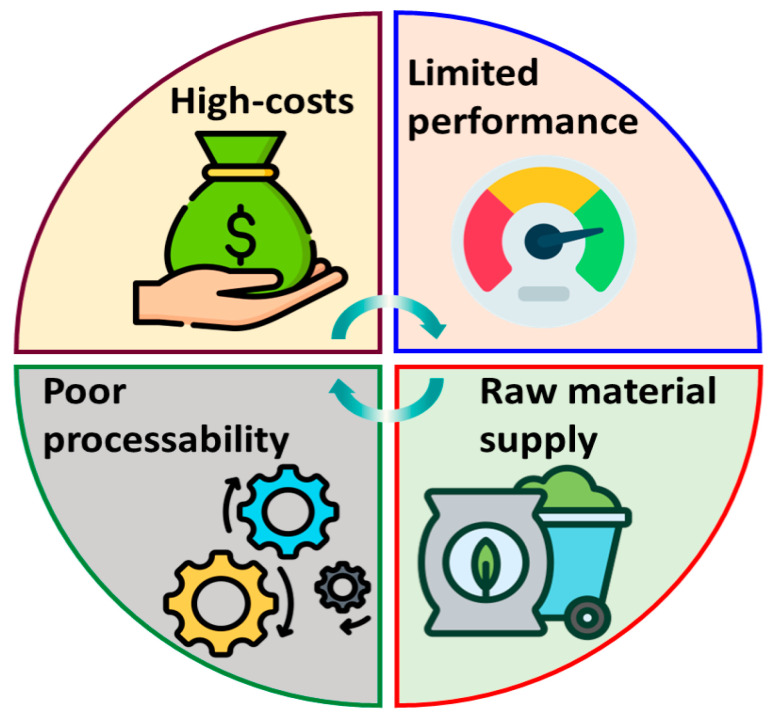
Major challenges in advancing the development of biopolymer-based packaging.

The machinability of the biopolymer-based packaging is an additional major challenge. Due to their mechanical strength, flexibility, and thermal stability, these materials are often unsuitable for industrial manufacturing processes like extrusion, molding, and printing [8,9]. These technological challenges need to be addressed in order to scale up production and meet packaging industry standards. By producing composites or mixing biopolymers with suitable additives, the machinability of these cutting-edge materials can be enhanced while maintaining their sustainable characteristics. A review of the main challenges preventing the advancement of biopolymeric film development can be seen in Figure 1. This review highlights the key challenges, summarizes the potential solutions, and future research directions to promote these sustainable and feasible biopolymer-based packaging structures. This review article also describes the challenges and possible solutions associated with food packaging materials. It examines the current state of research and industrial application, including the advantages and disadvantages of various biopolymer-based food packaging [10].

## 2. Recent Developments in Biopolymer-Based Films

Biodegradable packaging has emerged as a formidable solution to combat waste-related challenges, offering multifaceted benefits [11]. Edible films strengthen their overall quality by enhancing the mechanical and structural integrity properties of these items [12,13,14,15]. Comprising a blend of various components, including polysaccharides, proteins, and lipids, or their combinations, edible and biodegradable biopolymers assume the form of a thin and transparent layer [16]. This layer delicately envelops the food without disturbing its original composition or processing methods. Significantly, this approach can maintain crucial attributes such as moisture control, gas barrier functions (including oxygen and CO_2_), sensory appeal, and overall appearance [17]. Furthermore, these biopolymers are a formidable barrier against microbial spoilage, enhancing food product longevity [18,19,20]. Biopolymers offer a promising avenue to curtail conventional packaging demand and reduce waste [21]. By extending the shelf life of food products and enhancing the economic efficiency of packaging materials, these solutions mark a substantial step toward sustainability [22]. Biopolymers stand as an eco-friendly alternative to synthetic plastics in food packaging. [23,24]. Within this framework, natural constituents such as polysaccharides, proteins, and lipids are harmoniously combined with plasticizers (glycerol, glycol, and polyol) and surfactants to cast a biopolymer [25]. The structure of biopolymers is established in their nontoxic, biodegradable, and environmentally friendly composition, derived from simple derivatives of plants and animals [26,27]. Various components can be applied to develop biodegradable films. The recognized polysaccharides include chitosan, pullulan, alginate, carrageenan, cellulose, starch, pectin, and diverse gums [28]. Commonly utilized options in the group of proteins include collagen, gelatin, corn zein, wheat gluten, soy protein, casein, and mung bean protein [29]. These bioplastics have as raw materials polymers like chitosan, starch, alginates, and proteins. They also include antimicrobial agents such as essential oils, antioxidants based on phenolic compounds, tocopherols and flavonoids, enzymes like lysozyme and glucose oxidase, and a series of plant extracts, like green tea, rosemary, turmeric, etc. Biopolymers also have applications in medicine and pharmaceutics for wound healing and controlled delivery systems. However, despite these scientific advances, a series of challenges still remained in their transfer to production at an industrial scale and commercialization [30].

**Figure 2:**
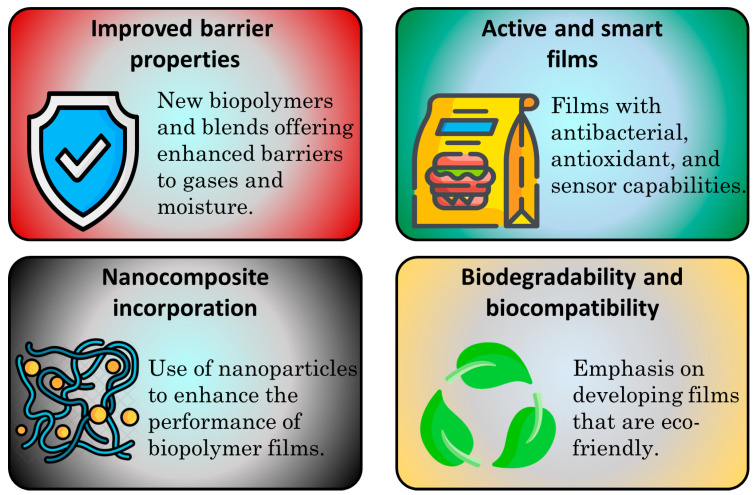
Recent developments of biopolymer-based films.

Recent developments in biopolymer-based films for food packaging have shown significant promise in reducing reliance on synthetic plastics. The recent developments in biopolymer films are shown in Figure 2. These films are derived from natural, renewable resources such as starch, cellulose, chitosan, and proteins like gelatin or soy. Innovations in formulation techniques, including blending and chemical modifications, have enhanced their physical properties, such as strength, flexibility, and moisture resistance. These advancements have allowed biopolymer films to increasingly rival traditional plastic films across a wide range of food packaging applications. Nanotechnology has played a vital role in advancing the performance of biopolymer-based films. The incorporation of nanomaterials such as nanocellulose, silver nanoparticles, or nano-clays has improved the barrier, antimicrobial, and mechanical properties of the films. These enhancements contribute to extended shelf life and improved food safety by preventing microbial growth and controlling gas exchange. Researchers are also exploring smart packaging technologies, where biopolymer films can respond to environmental stimuli or indicate product freshness.

Biopolymer-based films have been identified as a viable alternative to traditional plastic materials, taking into consideration the environmental consequences of their non-biodegradable behavior. These bioplastics are based on natural polymers (chitosan, starch, alginates, and proteins) that offer biodegradability and functional properties for a diversity of applications, mainly in food packaging. These include active films containing natural components that can enhance food preservation, like biopolymer matrix-based films that facilitate a controlled release of bioactive compounds [31,32,33]. Natural compounds contained in active food packaging includes antimicrobial agents (essential oils, nisin, bacteriocins), antioxidants (phenolic compounds, tocopherols, flavonoids), enzymes (lysozyme, glucose oxidase), bioactive peptides, and plant extracts (including green tea, rosemary, turmeric), which help in extending shelf life, improving safety, and preserving food quality [34,35,36,37].

Intelligent films using pH-sensitive dyes have been developed for smart packing, achieving real-time monitoring of food freshness [38,39]. Furthermore, the limitations of biopolymers can be mitigated through the incorporation of nanofillers. These nanocomposite films present beneficial applications in active food packaging due to their improved mechanical properties, water vapor barrier properties, and wide antimicrobial activity [40,41]. Apart from packaging applications, biopolymer films are also gaining attention in medicine and pharmaceuticals, with applications like wound healing and controlled delivery systems. Films are formulated with bioactive compounds or customized drug-release properties for use in dentistry, surgery, and dermatology [42]. Also, smart biopolymer films incorporated with antimicrobial agents and antioxidants have shown their effectiveness in extending food shelf life and safety. Despite these innovations, challenges, including high production costs, poor mechanical and barrier properties, complex migration kinetics, as well as potential toxicity and processability, still require further optimization of formulations and processing methods. Despite these advances, challenges remain in the scalability, cost, and regulatory approval of biopolymer-based films. Continuous research is being directed toward improving production efficiency and developing hybrid films that combine the benefits of different biopolymers.

## 3. Properties of Biopolymer-Based Packaging

Biopolymer-based packaging materials exhibit a wide range of physicochemical and functional properties that determine their suitability as alternatives to conventional plastics in food packaging. These properties depend largely on the type of biopolymer (e.g., polysaccharides, proteins, or polyesters) and the incorporation of additives, plasticizers, or nanomaterials [5].

### 3.1. Mechanical Properties

The mechanical properties of biopolymer-based packaging materials, such as tensile strength, elasticity, and flexibility, play a critical role in determining their performance and suitability for food preservation [43,44,45]. Many biopolymers, including polysaccharides (starch, chitosan, alginates) and proteins (gelatin, soy protein, casein), exhibit good film-forming ability and transparency, but they often lack the mechanical robustness of conventional plastics like polyethylene or polypropylene. These natural polymers are generally brittle, with low elongation at break and moderate tensile strength, which can limit their use in demanding packaging applications that require high durability and resistance to handling stresses. Plasticizers such as glycerol or sorbitol are commonly added to improve flexibility, while blending with other biopolymers can enhance overall strength and ductility [46]. To further address these limitations, recent advancements have explored reinforcement strategies through nanotechnology and hybrid materials. The incorporation of nanofillers, such as nanoclays, cellulose nanofibers, silica, or metal oxide nanoparticles, has been shown to significantly improve tensile strength, toughness, and barrier properties by creating strong interfacial interactions within the polymer matrix. Similarly, multilayer structures combining different biopolymers or biopolymer–synthetic polymer composites can balance flexibility and strength [47]. Despite these improvements, achieving consistent mechanical performance on a large industrial scale remains a challenge, particularly due to variability in raw material sources and the sensitivity of natural polymers to humidity. Thus, optimizing the mechanical properties of biopolymer-based packaging remains a key research focus for enabling their widespread commercial adoption [48,49].

### 3.2. Barrier Properties

Barrier properties, particularly against gases (oxygen, carbon dioxide) and water vapor, are crucial for determining the effectiveness of packaging materials in preserving food quality and extending shelf life [50]. Biopolymers such as polysaccharides (starch, cellulose derivatives, chitosan) and proteins (gelatin, soy protein, casein) generally exhibit excellent gas barrier properties due to their dense molecular structure and strong intermolecular interactions, which hinder the diffusion of oxygen and carbon dioxide. This makes them highly suitable for packaging oxygen-sensitive foods, where maintaining low oxygen permeability is essential to prevent lipid oxidation, color changes, and microbial spoilage. Some polyesters, such as polylactic acid (PLA) and polyhydroxyalkanoates (PHA), also provide reasonable gas barrier properties and are already used in commercial packaging applications [51]. However, a major limitation of most biopolymer-based packaging materials is their poor water vapor resistance, primarily due to their hydrophilic nature. High moisture permeability reduces their effectiveness in packaging high-moisture or humidity-sensitive foods. To overcome this drawback, several strategies have been explored, including chemical modifications (e.g., acetylation, cross-linking), blending with hydrophobic polymers, surface coatings, and the incorporation of nanofillers such as nanoclays, silica nanoparticles, or cellulose nanocrystals. These modifications can create more tortuous pathways for water molecules, thereby improving moisture barrier performance. Despite these advancements, achieving an optimal balance between gas and water vapor barrier properties remains challenging, especially under varying storage conditions, and continues to be a key area of research for the industrial application of biopolymer-based packaging [52,53,54].

### 3.3. Functional Properties (Active and Smart Packaging)

Beyond serving as passive barriers, biopolymer-based packaging systems can be designed with active functions to enhance food preservation and safety [55]. Active packaging incorporates bioactive compounds such as antimicrobial agents (e.g., essential oils, silver nanoparticles, lysozyme), antioxidants (e.g., polyphenols, tocopherols, flavonoids), or enzymes that help delay spoilage and extend shelf life. For example, chitosan-based films possess inherent antimicrobial properties, while starch or protein films can be functionalized with plant extracts such as green tea, rosemary, or turmeric to provide antioxidant activity [56]. These active components not only protect food against microbial contamination and oxidative degradation but also reduce the need for synthetic chemical preservatives, aligning with consumer demand for natural and “clean-label” food packaging. In addition, smart packaging systems based on biopolymers are being developed to monitor food quality in real time. Smart films can incorporate pH-sensitive dyes, biosensors, or colorimetric indicators that change color in response to spoilage-related changes such as gas release, microbial growth, or pH shifts. For instance, anthocyanin-loaded biopolymer films can act as freshness indicators by exhibiting visible color changes as food deteriorates. Such innovations allow consumers and retailers to assess food safety directly, reducing food waste and improving supply chain management [57,58]. Despite these advances, challenges remain in ensuring the stability, safety, and regulatory approval of active components and indicators. Nevertheless, active and smart biopolymer-based packaging represents a promising next generation of sustainable materials that can simultaneously extend shelf life, improve safety, and enhance consumer confidence.

### 3.4. Biodegradability and Composability

One of the most significant advantages of biopolymer-based packaging materials is their inherent biodegradability, which offers a sustainable alternative to persistent petroleum-derived plastics. Biopolymers such as starch, cellulose, chitosan, proteins, polylactic acid (PLA), and polyhydroxyalkanoates (PHA) can be broken down by naturally occurring microorganisms into carbon dioxide, water, methane (under anaerobic conditions), and biomass, thereby reducing long-term environmental pollution [59]. This degradability helps to mitigate plastic accumulation in landfills and marine environments, aligning with global efforts toward waste reduction and circular economy practices. Furthermore, many biopolymers are also compostable, meaning they can decompose under controlled conditions in industrial composting facilities, generating nutrient-rich compost that can be used to improve soil health. However, the biodegradability and compostability of biopolymer packaging depend strongly on the material type and the environmental conditions in which disposal occurs. For example, while starch- and protein-based films degrade relatively quickly under ambient conditions, PLA often requires elevated temperatures and humidity typically found in industrial composting facilities to break down effectively. Similarly, multilayer biopolymer films or those reinforced with nanomaterials may show slower degradation rates. The lack of standardized global composting infrastructure poses additional challenges, as “compostable” materials may not degrade properly in natural environments, potentially leading to consumer confusion. Therefore, while biodegradability and composability provide clear environmental benefits, further research and regulatory support are needed to optimize formulations and ensure proper end-of-life management for large-scale adoption.

### 3.5. Complex Migration Kinetics

**Figure 3:**
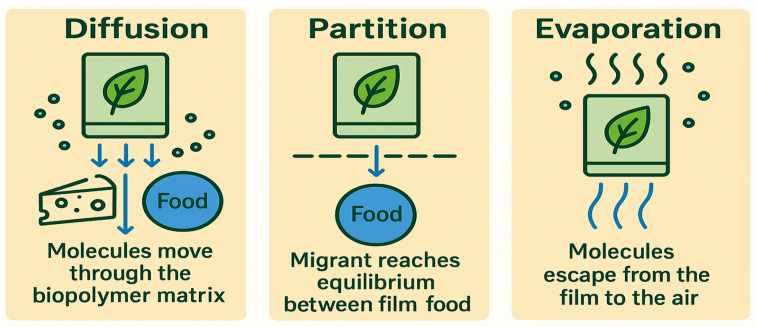
Migration mechanism of biopolymer-based film for packaging.

Complex migration kinetics refers to the process in which certain substances move or transfer from packaging materials into the food or the surrounding environment. Factors affecting this process include the structural properties of the biopolymer, the release behavior of the migrating substances, and the kinetic models that describe their movement. At the core of this process is a migration matrix representing the physical and chemical structure of the biopolymer (Figure 3). For instance, polymers with high molecular crystallinity, such as polylactic acid (PLA) and polybutylene succinate (PBS), and poly(butylene adipate-*co*-terephthalate) (PBAT), develop tightly packed structures resulting in slow diffusion. This happens as the increased crystallinity restricts molecular mobility, therefore acting as a barrier in the matrix [60]. The compatibility between the migrating substance and the biopolymer matrix is another important factor influencing migration rates. Hydrophilic materials, for example, diffuse more easily through hydrophilic matrices, while hydrophobic bonds can hold substances within the polymer, limiting their diffusion [61].

Migration behavior is further complicated by the development of the packaging material, such as factors like porosity and the addition of ingredients such as nanoparticles. Nanoparticles are widely used to enhance the barrier properties of biopolymers through reduced permeability, although they may also pose a risk through migration into food themselves [62]. Zero-order kinetics is commonly used in controlled-release applications to ensure a constant migration rate throughout time. This strategy is particularly advantageous for active packaging systems, where antimicrobials or antioxidants are continuously released to preserve food quality. Under zero-order kinetics, the release rate remains constant, ensuring uniform delivery and prolonged effectiveness without fluctuations in concentration. These controlled-release mechanisms may be tailored toward specific needs by controlling the physiology of the polymer matrix or adding functional additives [63,64].

Migration is mainly a diffusion process depending on the concentration gradient between the packaging (film) and food or simulants. Molecular size, temperature, and the chemical affinity of the migrant with the polymer matrix can affect diffusion rates. Increased temperature, for example, raises molecular motion and enhances diffusion, while the combination of smaller molecules and greater compatibility promotes higher migration [65]. However, modeling the migration kinetics of biopolymer-based packaging remains challenging. The migration behavior of the many different substances with varying diffusion rates is often difficult to predict accurately, especially for changing environmental conditions like temperature, humidity, and food composition. Such factors can substantially alter migration dynamics and the behavior of leachable compounds, making standardization for regulatory purposes challenging. Furthermore, interactions between biopolymers and leachable substances add to the complexity of predictions for emerging materials, including bio-based nanocomposites. The behavior of such novel materials in the context of migrating in foods is poorly understood and therefore limits their functionality, relying on further studies to fill the gaps in the available scientific knowledge [66].

### 3.6. Toxicity

Biopolymer-based packaging materials are generally considered safer and more environmentally friendly alternatives to conventional plastics; however, concerns regarding their potential toxicity have emerged. These materials, derived from natural sources such as starch, cellulose, and proteins, may still contain or release harmful substances, including residual monomers, plasticizers, or additives used to enhance performance. Additionally, during degradation or interaction with food products, biopolymers can form byproducts that may pose health risks. The transition toward biopolymer-based packaging is a result of the desire for environmental sustainability and the replacement of traditional petroleum-based plastics. However, the potential toxicity of biopolymers—particularly related to migration behaviors, degradation products, and interactions with food—remains a critical issue that requires thorough investigation. Biopolymers are frequently supplemented with additives to improve their mechanical, thermal, and barrier properties. However, these additives can leach into food, raising concerns about toxicity. As an example, different studies pointed out that adding nanoparticles to biopolymer matrices improved packaging characteristics, but they can migrate into the food, which could expose the consumer to health risks due to their unknown effects on human beings over the long term [67]. Additionally, oligomers and unreacted monomers from biopolymers like polylactic acid (PLA) have been found in food simulants, requiring their migration levels to be stringently controlled [68].

**Figure 4:**
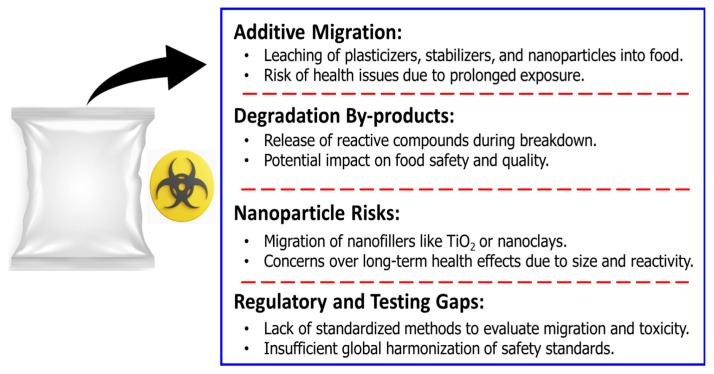
Toxicity concerns of biopolymer-based packaging material.

Biopolymers can also degrade under heat, humidity, or light exposure, releasing substances that have an impact on food safety. The heat-induced degradation of biopolymers such as PLA generates acids, which lower the pH of the food matrix and can react with the food components [69]. Bioactive compounds, including essential oils and natural pigments, are commonly integrated into active and smart biopolymer packages to provide antimicrobial or freshness-monitoring functions. Although most of these additives are safer than synthetic chemicals, their migration into food must be controlled in order to reduce their effect on the sensory properties of food or the introduction of these substances beyond their acceptable limits. To improve barrier and mechanical properties, nanotechnology-enhanced biopolymers, such as clay or titanium dioxide-reinforced films, are being increasingly developed. However, the high reactivity and small size of nanoparticles pose a risk for toxicity upon migration. There is a need for in-depth toxicological evaluation of these materials to confirm that they remain within safety parameters [70]. While the use of biopolymers is on the rise, regulatory frameworks for biopolymer safety continue to evolve. It is necessary to establish standardized procedures for testing migration, degradation products, and interactions with food to ensure compliance with safety standards. In addition, life-cycle assessments are necessary to assess their overall environmental and health impacts [1]. Figure 4 lists the main issues associated with the toxicity of biopolymer-based packaging materials. The toxicity concerns with different biopolymer-based packaging materials are presented in Table 1.

## 4. Environmental Impact of Biopolymer Packaging

Biopolymer-based packaging materials have emerged as a sustainable alternative to conventional petroleum-derived plastics due to their biodegradability, renewable origin, and reduced carbon footprint [71]. Unlike synthetic plastics, which persist in the environment for centuries, biopolymers such as polysaccharides, proteins, polylactic acid (PLA), and polyhydroxyalkanoates (PHA) can undergo natural degradation processes, minimizing long-term pollution. Moreover, their production from agricultural by-products and renewable feedstocks contributes to circular economy principles and waste valorization. However, the environmental benefits of biopolymer packaging are not absolute. Large-scale production may compete with food resources, raising concerns about land use, water consumption, and agricultural sustainability. Additionally, the energy demand for processing some biopolymers (e.g., PLA) may still be significant compared to traditional plastics [72,73]. End-of-life management also remains a challenge, as biodegradability is strongly dependent on environmental conditions—many biopolymers require industrial composting facilities to degrade effectively, which are not universally available. Therefore, while biopolymer packaging reduces environmental burden, its full impact should be evaluated through comprehensive life cycle assessments (LCA) to balance sustainability with scalability [74].

## 5. Human Health Implications of Biopolymer Packaging

The safety of food-contact materials is a critical consideration in biopolymer packaging. Many biopolymers are inherently biocompatible and non-toxic, which makes them suitable for direct contact with food products [75]. Their natural origin reduces the risk of releasing harmful synthetic additives or microplastics into food, which are growing concerns with conventional plastics. Moreover, the incorporation of bioactive agents (e.g., antimicrobial or antioxidant compounds) into active packaging systems may extend shelf life while ensuring food safety [76]. Nevertheless, certain challenges remain. Migration of residual monomers, plasticizers, or incorporated nanoparticles into food matrices poses potential toxicity risks and requires rigorous safety evaluation. For example, while nanomaterials such as ZnO [77], TiO_2_ [78], or Ag nanoparticles [61,79] can improve antimicrobial and barrier properties, their uncontrolled migration may lead to health concerns due to bioaccumulation. Furthermore, degradation products of some biopolymers under high temperature or humidity conditions may alter food quality or safety. Regulatory frameworks, including those from the U.S. FDA and EFSA, emphasize the need for systematic risk assessment, migration studies, and compliance testing before large-scale commercialization. Therefore, while biopolymer packaging offers promising benefits for human health compared to petroleum-based plastics, ensuring food safety through careful material selection, controlled incorporation of additives, and adherence to regulatory standards remains essential.

## 6. Application of Biodegradable Packaging in the Industry

**Figure 5:**
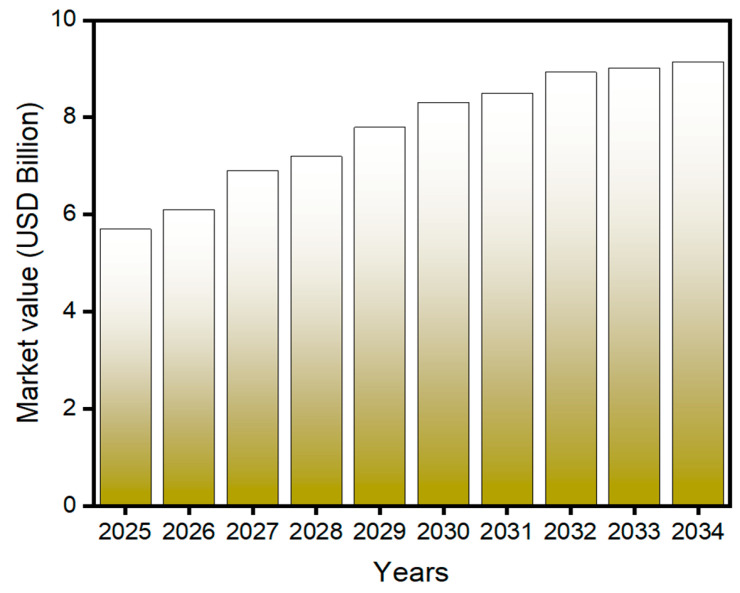
Forecasted Global Market Size of Biodegradable Packaging from 2025 to 2034 (in USD 975 Billion.

According to Business Research Insights (2025) [80], The global biodegradable packaging market was valued at approximately USD 5.70 billion in 2025 and is projected to grow to USD 9.15 billion by 2034, reflecting a compound annual growth rate (CAGR) of 6.05% over the forecast period from 2025 to 2034 (Figure 5). Biodegradable packaging refers to materials designed for packaging applications that can naturally break down into non-toxic components through the action of microorganisms such as bacteria and fungi. These materials are specifically developed to reduce environmental impact and minimize the accumulation of persistent, non-degradable waste [80,81]. The COVID-19 pandemic has significantly increased the global awareness of environmental concerns, especially the impact of plastic pollution. This growing consciousness has intensified the emphasis on sustainable alternatives, leading to a rising interest in and demand for biodegradable packaging solutions. Biodegradable packaging is utilized across multiple industries, including food and beverage, pharmaceuticals, home care, cosmetics, and other consumer goods. In the food and beverage sectors. In pharmaceutical and biomedical fields, it is used to safely package medications and medical devices, meeting strict regulatory standards while reducing environmental impact. For home care and cosmetics, biodegradable materials are increasingly preferred for packaging personal care products and household cleaners, aligning with consumer demand for sustainable alternatives. Additionally, biodegradable packaging is adopted in sectors such as electronics and textiles, demonstrating its versatility and growing relevance in sustainable product design [82].

## 7. Scaling Up Challenges

The use of biopolymer-based packaging materials is a promising route to reduce the environmental impact, but their continued development faces major challenges to scalability. Overcoming these challenges across machinability, mechanical performance, sourcing of raw materials, and market acceptance is necessary to replace traditional plastic materials. A significant hurdle is the machinability of biopolymers in high-speed manufacturing systems. Unlike conventional plastics, however, biopolymers often do not possess the necessary flow properties and thermal stability to effectively work in existing extrusion, molding, and thermoforming processing equipment. This can create inefficiencies and defects during production. Various approaches have been examined for the mixing and integration of biopolymers with plasticizers or additives to improve their processability, but usually at the expense of mechanical strength or biodegradability [83]. The other most important and critical aspect is improving the mechanical strength, moisture resistance, and barrier properties of biopolymers comparable to conventional plastics. Most biopolymers tend to be brittle, have good barrier properties to water but poor barriers to oxygen, further limiting their application in food packaging and other fields. These drawbacks have been addressed by developing new biocomposite materials such as reinforced biopolymers with nanofillers like cellulose nanocrystals or graphene oxide. Nonetheless, these methods increase production complexity and costs [84]. Another challenge in scaling up biopolymer-based packaging is its higher production costs. Biopolymers typically involve higher-cost raw materials and processing methods, resulting in increased production costs. Moreover, consumer and industry hesitation further leads to limited market acceptance. Educating stakeholders and representing the benefits associated with biopolymer adaptation, such as environmental gains and reduction in regulatory issues, could remove these obstacles [5]. Table 2 provides a comprehensive summary of the limitations hindering the development of biopolymer-based films in applications. Recent advancements in biopolymer-based food packaging have made significant strides toward sustainability and functionality. Researchers are exploring various materials and technologies to address environmental concerns associated with traditional plastic packaging [85,86,87].

## 8. Conclusions

Packaging materials based on biopolymers exhibit the promise as an alternative to conventional plastic packaging materials in addressing environmental pollution. Nonetheless, the successful adoption of biopolymers in the packaging industry hinges on overcoming key challenges, such as inadequate mechanical and barrier properties, restricted processability, and high production costs. Biopolymer-based packaging represents a promising pathway toward sustainable and eco-friendly alternatives to conventional petroleum-derived plastics. The present review highlights significant progress in developing diverse biopolymer systems with enhanced barrier, mechanical, and functional properties. Recent innovations, including active and smart packaging and the incorporation of nanomaterials, further extend their potential applications in the food industry. Despite these advancements, several challenges continue to limit large-scale adoption. Issues such as limited machinability, relatively high production costs, and dependency on specific environmental conditions for biodegradation remain critical barriers. Additionally, ensuring consistent material performance, minimizing migration risks, and addressing regulatory and consumer acceptance concerns are essential for successful commercialization. Future research should focus on low-cost and scalable production methods, optimization of mechanical and barrier properties, safe integration of functional additives, and comprehensive life cycle assessments to validate environmental benefits. Collaboration between researchers, industry stakeholders, and policymakers will be vital to accelerating the transition from conventional plastics to next-generation biopolymer packaging. In conclusion, while biopolymers are not yet a complete replacement for plastics, they represent an essential step toward sustainable packaging solutions. With continued scientific and technological advancements, they hold the potential to reshape the future of food packaging and contribute to global environmental sustainability.

## Figures and Tables

**Table 1:** Toxicity of biopolymer-based packaging materials.

S. No	Biopolymer	Source	Toxicity Concerns	Toxic By-Products	Toxicity Assessment
1.	Polylactic Acid (PLA)	Corn starch, sugarcane	Generally considered safe; some concerns with degradation by-products under heat	Lactide monomer, additives like plasticizers	Low toxicity; FDA-approved for food contact
2.	Polyhydroxyalkanoates (PHA)	Bacterial fermentation of sugars	Biocompatible and biodegradable; minimal toxicity	Require plasticizers or solvents in process	Generally non-toxic; safe for medical applications
3.	Starch-based polymers	Corn, potato, wheat starch	Potential for microbial contamination and degradation products	Glycerol (plasticizer), residual solvents	Low toxicity if properly processed
4.	Cellulose derivatives	Wood pulp, cotton linters	Non-toxic, biodegradable; degradation by-products are not harmful	Acetylation chemicals (for cellulose acetate)	Non-toxic; widely used in pharmaceutical coatings
5.	Chitosan	Chitin (shellfish exoskeleton)	Generally non-toxic; potential allergen for people with shellfish allergies	Acidic solvents used in preparation	Low toxicity, but allergenicity is a concern
6.	Gelatin-based films	Animal collagen	Biodegradable and non-toxic; microbial growth if not properly preserved	Cross-linking agents (e.g., glutaraldehyde)	Safe if additives are controlled
7.	Protein-based films	Soy, whey, casein	Biodegradable; risk of allergenicity or microbial degradation	Cross-linkers or preservatives	Generally safe; allergenic potential must be assessed
8.	Alginates	Seaweed	Low toxicity; widely used in food and pharma	Calcium salts (used in gelation)	Safe for ingestion; FDA-approved
9.	Agar	Red algae	Safe and widely used in food packaging	No harmful additives	Non-toxic
10.	Carrageenan	Red seaweed	Some types linked to gastrointestinal inflammation in high doses (e.g., degraded form)	Processing residues	Food-grade form is considered safe in moderation

**Table 2:** Different challenges in advancing the biopolymer-based packaging in the food market.

Challenge Category	Key Issues	Details	References
Release kinetics	Inconsistent compound release	Difficulty in achieving controlled, uniform release of active compounds for food preservation	[83]
	Substance migration	Migration of plasticizers, nanoparticles, and other additives into food poses safety risks	[84]
Toxicity	Additive leaching	Substances like stabilizers and antimicrobial agents can leach into food, impacting health and safety	[35]
	Byproduct release	Degradation of biopolymers may release reactive or acidic compounds harmful to food and humans	[7]
	Nanoparticle migration	Nanofillers like titanium dioxide may migrate into food, raising health concerns	[88]
Machinability	Thermal stability issues	Poor thermal properties hinder use in extrusion, molding, and high-speed industrial processes	[89]
	Mechanical limitations	Brittleness and low mechanical strength restrict biopolymer applications	[90]
	Compatibility issues	Difficult to adapt biopolymers to existing manufacturing systems	[91]

## Data Availability

No new data were created or analyzed in this study.

## References

[1] Sinha S. (2024). An overview of biopolymer-derived packaging material. Polym. Renew. Resour..

[2] Asgher M., Qamar S.A., Bilal M., Iqbal H.M.N. (2020). Bio-based active food packaging materials: Sustainable alternative to conventional petrochemical-based packaging materials. Food Res. Int..

[3] Ahmed M.W., Haque M.A., Mohibbullah M., Khan M.S.I., Islam M.A., Mondal M.H.T., Ahmmed R. (2022). A review on active packaging for quality and safety of foods: Current trends, applications, prospects and challenges. Food Packag. Shelf Life.

[4] Eyiz V., Tontul I. (2021). Bioactive components structure and their applications in active packaging for shelf stability enhancement. Active Packaging for Various Food Applications.

[5] Perera K.Y., Jaiswal A.K., Jaiswal S. (2023). Biopolymer-based sustainable food packaging materials: Challenges, solutions, and application. Foods.

[6] Zhang M., Zuo Z., Zhang X., Wang L. (2024). Food biopolymer behaviors in the digestive tract: Implications for nutrient delivery. Crit. Rev. Food Sci. Nutr..

[7] Pinaeva L.G., Noskov A.S. (2024). Biodegradable biopolymers: Real impact to environment pollution. Sci. Total Environ..

[8] Donkor L., Kontoh G., Yaya A., Bediako J.K., Apalangya V. (2023). Bio-based and sustainable food packaging systems: Relevance, challenges, and prospects. Appl. Food Res..

[9] Stoica M., Bichescu C.I., Crețu C.-M., Dragomir M., Ivan A.S., Podaru G.M., Stoica D., Stuparu-Crețu M. (2024). Review of bio-based biodegradable polymers: Smart solutions for sustainable food packaging. Foods.

[10] Zhang L., Yu D., Regenstein J.M., Xia W., Dong J. (2021). A comprehensive review on natural bioactive films with controlled release characteristics and their applications in foods and pharmaceuticals. Trends Food Sci. Technol..

[11] Petkoska A.T., Daniloski D., D’Cunha N.M., Naumovski N., Broach A.T. (2021). Edible packaging: Sustainable solutions and novel trends in food packaging. Food Res. Int..

[12] Kumar N., Neeraj (2019). Polysaccharide-based component and their relevance in edible film/coating: A review. Nutr. Food Sci..

[13] Kumar N., Pratibha, Prasad J., Yadav A., Upadhyay A., Neeraj, Shukla S., Petkoska A.T., Heena, Suri S. (2023). Recent trends in edible packaging for food applications—Perspective for the future. Food Eng. Rev..

[14] Khwaldia K., Perez C., Banon S., Desobry S., Hardy J. (2004). Milk proteins for films and coatings. Crit. Rev. Food Sci. Nutr..

[15] Nilsen-Nygaard J., Fernández E.N., Radusin T., Rotabakk B.T., Sarfraz J., Sharmin N., Sivertsvik M., Sone I., Pettersen M.K. (2021). Current status of biobased and biodegradable food packaging materials: Impact on food quality and effect of innovative processing technologies. Compr. Rev. Food Sci. Food Saf..

[16] Falguera V., Quintero J.P., Jiménez A., Muñoz J.A., Ibarz A. (2011). Edible films and coatings: Structures, active functions and trends in their use. Trends Food Sci. Technol..

[17] Qiu L., Zhang M., Tang J., Adhikari B., Cao P. (2019). Innovative technologies for producing and preserving intermediate moisture foods: A review. Food Res. Int..

[18] Chettri S., Sharma N., Mohite A.M. (2023). Edible coatings and films for shelf-life extension of fruit and vegetables. Biomater. Adv..

[19] Zhao Y., Zhang Y., Dong H., Wu W., Yang X., He Q. (2023). Functional biopolymers for food packaging: Formation mechanism and performance improvement of chitosan-based composites. Food Biosci..

[20] Periyasamy T., Asrafali S.P., Lee J. (2025). Recent advances in functional biopolymer films with antimicrobial and antioxidant properties for enhanced food packaging. Polymers.

[21] Chawla R., Sivakumar S., Kaur H. (2021). Antimicrobial edible films in food packaging: Current scenario and recent nanotechnological advancements- A review. Carbohydr. Polym. Technol. Appl..

[22] Mezemir S.A., Solomon W.K. (2017). Effect of bee wax and linseed oil coatings and frequency of dipping on the biochemical and organoleptic quality of fresh orange juice (*Citrus sinensis* cv. Valencia). J. Postharvest Technol..

[23] Gaspar M.C., Braga M.E.M. (2023). Edible films and coatings based on agrifood residues: A new trend in the food packaging research. Curr. Opin. Food Sci..

[24] Prameela K., Mohan C.M., Ramakrishna C., Holban A.M. (2018). Biopolymers for food design: Consumer-friendly natural ingredients. Biopolymers for Food Design.

[25] Siracusa V., Rocculi P., Romani S., Rosa M.D. (2008). Biodegradable polymers for food packaging: A review. Trends Food Sci. Technol..

[26] Teixeira-Costa B.E., Andrade C.T. (2021). Natural polymers used in edible food packaging—History, function and application trends as a sustainable alternative to synthetic plastic. Polysaccharides.

[27] Samir A., Ashour F.H., Hakim A.A.A., Bassyouni M. (2022). Recent advances in biodegradable polymers for sustainable applications. npj Mater. Degrad..

[28] Karnwal A., Kumar G., Singh R., Selvaraj M., Malik T., Al Tawaha A.R.M. (2024). Natural biopolymers in edible coatings: Applications in food preservation. Food Chem. X.

[29] Coltelli M.-B., Wild F., Bugnicourt E., Cinelli P., Lindner M., Schmid M., Weckel V., Müller K., Rodriguez P., Staebler A. (2015). State of the art in the development and properties of protein-based films and coatings and their applicability to cellulose based products: An extensive review. Coatings.

[30] Das A., Ringu T., Ghosh S., Pramanik N. (2023). A comprehensive review on recent advances in preparation, physicochemical characterization, and bioengineering applications of biopolymers. Polym. Bull..

[31] Benbettaïeb N., Karbowiak T., Debeaufort F. (2019). Bioactive edible films for food applications: Influence of the bioactive compounds on film structure and properties. Crit. Rev. Food Sci. Nutr..

[32] Chen W., Ma S., Wang Q., McClements D.J., Liu X., Ngai T., Liu F. (2022). Fortification of edible films with bioactive agents: A review of their formation, properties, and application in food preservation. Crit. Rev. Food Sci. Nutr..

[33] Deshmukh R.K., Gaikwad K.K. (2022). Natural antimicrobial and antioxidant compounds for active food packaging applications. Biomass Convers. Biorefin..

[34] Duda-Chodak A., Tarko T., Petka-Poniatowska K. (2023). Antimicrobial compounds in food packaging. Int. J. Mol. Sci..

[35] Vasile C., Baican M. (2021). Progresses in food packaging, food quality, and safety—Controlled-release antioxidant and/or antimicrobial packaging. Molecules.

[36] Singh A.K., Kim J.Y., Lee Y.S. (2022). Phenolic compounds in active packaging and edible films/coatings: Natural bioactive molecules and novel packaging ingredients. Molecules.

[37] Alizadeh-Sani M., Mohammadian E., Rhim J.-W., Jafari S.M. (2020). pH-sensitive (halochromic) smart packaging films based on natural food colorants for the monitoring of food quality and safety. Trends Food Sci. Technol..

[38] Liu D., Zhang C., Pu Y., Chen S., Liu L., Cui Z., Zhong Y. (2022). Recent advances in pH-responsive freshness indicators using natural food colorants to monitor food freshness. Foods.

[39] Jamróz E., Kulawik P., Kopel P. (2019). The effect of nanofillers on the functional properties of biopolymer-based films: A review. Polymers.

[40] Dharini V., Selvam S.P., Jayaramudu J., Emmanuel R.S. (2022). Functional properties of clay nanofillers used in the biopolymer-based composite films for active food packaging applications-Review. Appl. Clay Sci..

[41] Paczkowska-Walendowska M., Kulawik M., Kwiatek J., Bikiaris D., Cielecka-Piontek J. (2025). Novel applications of natural biomaterials in dentistry—Properties, uses, and development perspectives. Materials.

[42] Klonos P.A., Chronaki K., Vouyiouka S., Kyritsis A. (2024). Effects of high crystallinity on the molecular mobility in poly (lactic acid)-based microcapsules. ACS Appl. Polym. Mater..

[43] Adak B., Baidya S., Teramoto Y. (2025). Biopolymers and their nanocomposites coated paper-based high barrier and sustainable food packaging materials. Carbohydr. Polym..

[44] Cheng J., Gao R., Zhu Y., Lin Q. (2024). Applications of biodegradable materials in food packaging: A review. Alex. Eng. J..

[45] Singh S., Habib M., Rao E.S., Kumar Y., Bashir K., Jan S., Jan K. (2025). A comprehensive overview of biodegradable packaging films: Part I—Sources, additives, and preparation methods. Discov. Food.

[46] Dong Y., Li Y., Ma Z., Rao Z., Zheng X., Tang K., Liu J. (2023). Effect of polyol plasticizers on properties and microstructure of soluble soybean polysaccharide edible films. Food Packag. Shelf Life.

[47] Venkatesan R., Rajeswari N. (2017). ZnO/PBAT nanocomposite films: Investigation on the mechanical and biological activity for food packaging. Polym. Adv. Technol..

[48] Venkatesan R., Surya S., Suganthi S., Muthuramamoorthy M., Pandiaraj S., Kim S.-C. (2023). Biodegradable composites from poly(butylene adipate-*co*-terephthalate) with carbon nanoparticles: Preparation, characterization and performances. Eniviron. Res..

[49] Venkatesan R., Mayakrishnan M., Alrashed M.M., Kim S.-C. (2025). Recent advances in PBAT (nano) composite materials for food packaging: A comprehensive review. J. Appli. Polym. Sci..

[50] Mengozzi A., Carullo D., Bot F., Farris S., Chiavaro E. (2025). Functional properties of food packaging solutions alternative to conventional multilayer systems. J. Food Sci. Technol..

[51] Mayakrishnan V., Venkatesan R., Madhavan A.A. (2025). Development and characterization of antimicrobial nisin/MMT K10/AgNPs nanocomposite coatings on oxygen plasma surface-modified polypropylene for food packaging applications. Food. Bioprocess Technol..

[52] Dutta D., Sit N. (2024). A comprehensive review on types and properties of biopolymers as sustainable bio-based alternatives for packaging. Food Biomacromol..

[53] Selvam T., Rahman N.M.M.A., Olivito F., Ilham Z., Ahmad R., Wan-Mohtar W.A.A.Q.I. (2025). Agricultural waste-derived biopolymers for sustainable food packaging: Challenges and future prospects. Polymers.

[54] Venkatesan R., Alagumalai K., Jebapriya M., Dhilipkumar T., Almutairi T.M., Kim S.-C. (2024). Enhancing PBAT nanocomposite films: The impact of AgVO_3_ nanorods on mechanical, hydrophobicity, and antibacterial properties. Polym. Compos..

[55] Prasad J., Kumar N., Pratibha, Jaiswal R., Yadav A., Sharma S.P., Fawole O.A., Kaushik N. (2025). Biopolymer based composite packaging: A sustainable approach for fruits and vegetables preservation. Appl. Food Res..

[56] Ștefănescu B.E., Socaciu C., Vodnar D.C. (2022). Recent progress in functional edible food packaging based on gelatin and chitosan. Coatings.

[57] Olabode O., Kumar N., De D. (2025). Food loss and waste management in the retail food supply chain: Methods and framework to achieve environmental sustainability. J. Environ. Manage..

[58] Tadesse T., Ramírez-Rodríguez S., Rahmani D., Gezahegn T.W., Verherbrugghen H., Vermeulen A., Skourletis N., Roig J.M.G. (2025). Healthy but also traceable: Consumer choices for traits of food waste-reducing innovations. J. Clean. Prod..

[59] Nizamuddin S., Baloch A.J., Chen C., Arif M., Mubarak N.M. (2024). Bio-based plastics, biodegradable plastics, and compostable plastics: Biodegradation mechanism, biodegradability standards and environmental stratagem. Int. Biodeterior. Biodegradation.

[60] Jariyasakoolroj P., Chongcharoenyanon B., Wadaugsorn K. (2024). Kinetic migration of PBS and PBSA biopolymers prepared by cast film extrusion and biaxial stretching: A combined experimental and modeling approach. J. Appl. Polym. Sci..

[61] Venkatesan R., Rajeswari N., Tamilselvi A. (2018). Antimicrobial, mechanical, barrier, and thermal properties of bio-based poly (butylene adipate-co-terephthalate) (PBAT)/Ag_2_O nanocomposite films for packaging application. Polym. Adv. Technol..

[62] Ali M., Horikawa S., Venkatesh S., Saha J., Hong J.W., Byrne M.E. (2007). Zero-order therapeutic release from imprinted hydrogel contact lenses within in vitro physiological ocular tear flow. Asian J. Control Release.

[63] Souza V.G.L., Rodrigues P.F., Duarte M.P., Fernando A.L. (2018). Antioxidant migration studies in chitosan films incorporated with plant extracts. J. Renew. Mater..

[64] Wu X. (2024). Using Brownian model to study the effect of temperature on diffusion coefficient. Theor. Nat. Sci..

[65] Bhunia K., Sablani S.S., Tang J., Rasco B. (2013). Migration of chemical compounds from packaging polymers during microwave, conventional heat treatment, and storage. Compr. Rev. Food Sci. Food Saf..

[66] Rhim J.-W., Ng P.K.W. (2007). Natural biopolymer-based nanocomposite films for packaging applications. Crit. Rev. Food Sci. Nutr..

[67] Ubeda S., Aznar M., Alfaro P., Nerín C. (2019). Migration of oligomers from a food contact biopolymer based on polylactic acid (PLA) and polyester. Anal. Bioanal. Chem..

[68] Teixeira S., Eblagon K.M., Miranda F., RPereira M.F., Figueiredo J.L. (2021). Towards controlled degradation of poly(lactic) acid in technical applications. C.

[69] Alizadeh-Sani M., Mohammadian E., McClements D.J. (2020). Eco-friendly active packaging consisting of nanostructured biopolymer matrix reinforced with TiO_2_ and essential oil: Application for preservation of refrigerated meat. Food Chem..

[70] Taherimehr M., YousefniaPasha H., Tabatabaeekoloor R., Pesaranhajiabbas E. (2021). Trends and challenges of biopolymer-based nanocomposites in food packaging. Compr. Rev. Food Sci. Food Saf..

[71] Bagri F., Priyadarshi R., Pircheraghi G., Imani M., Rhim J.-W. (2025). Designing entirely biodegradable active food packaging using natural biopolymers and plant-derived additives: A cradle-to-cradle approach. Mater. Today Commun..

[72] Abang S., Wong F., Sarbatly R., Sariau J., Baini R., Besar N.A. (2023). Bioplastic classifications and innovations in antibacterial, antifungal, and antioxidant applications. J. Bioresour. Bioprod..

[73] Islam M., Xayachak T., Haque N., Lau D., Bhuiyan M., Pramanik B.K. (2024). Impact of bioplastics on environment from its production to end-of-life. Process Saf. Environ. Prot..

[74] Asim Z., Shahzad H.M.A., Ghodake G., Mahmoud K.A., Almomani F., Rasool K. (2025). Transforming agricultural food waste into bioplastics: Methods, potential, and technological advances. Adv. Sustain. Syst..

[75] Westlake J.R., Tran M.W., Jiang Y., Zhang X., Burrows A.D., Xie M. (2023). Biodegradable biopolymers for active packaging: Demand, development and directions. Sustainable Food Technol..

[76] Kadirvel V., Palanisamy Y., Ganesan N.D. (2025). Active packaging system—An overview of recent advances for enhanced food quality and safety. Pack. Tech. Sci..

[77] Yang J., Xiong D., Long M. (2025). Zinc oxide nanoparticles as next-generation feed additives: Bridging antimicrobial efficacy, growth promotion, and sustainable strategies in animal nutrition. Nanomaterials.

[78] Venkatesan R., Rajeswari N. (2017). TiO_2_ nanoparticles/poly(butylene adipate-*co*-terephthalate) bionanocomposite films for packaging applications. Polym. Adv. Technol..

[79] Istiqola A., Syafiuddin A. (2020). A review of silver nanoparticles in food packaging technologies: Regulation, methods, properties, migration, and future challenges. J. Chin. Chem. Soc..

[80] (2025). Business Research Insights. https://www.businessresearchinsights.com/market-reports/biodegradable-packaging-1128market-110971.

[81] Alaghemandi M. (2024). Sustainable solutions through innovative plastic waste recycling technologies. Sustainability.

[82] Ashiwaju B.I., Orikpete O.F., Fawole A.A., Alade E.Y., Odogwu C. (2023). A step toward sustainability: A review of biodegradable packaging in the pharmaceutical industry. Matrix Sci. Pharma.

[83] Khezerlou A., Tavassoli M., Alizadeh Sani M., Mohammadi K., Ehsani A., McClements D.J. (2021). Application of nanotechnology to improve the performance of biodegradable biopolymer-based packaging materials. Polymers.

[84] Thakur V., Satapathy B.K., Kumar S., Mukherjee A., Katiyar V. (2024). Migration concerns of biopolymer-based food packaging. Agro-Waste Derived Biopolymers and Biocomposites: Innovations and Sustainability in Food Packaging.

[85] Khan A.A., Ullah M.W., Qayum A., Khalifa I., Ul-Islam M., Bacha S.A.S., Zeb U., Yao F.-J., Alharbi S.A., Shrahili M. (2024). Structure-property relationship of ultrasound-assisted nanoemulsion-impregnated bioactive polysaccharide films for enhanced shelf life of mushrooms. Food Packag. Shelf Life.

[86] Khan A.A., Chi F.-J., Ullah M.W., Qayum A., Khalifa I., Bacha S.A.S., Ying Z.-Z., Khan I., Zeb U., Alarfaj A.A. (2024). Fabrication and characterization of bioactive curdlan and sodium alginate films for enhancing the shelf life of *Volvariella volvacea*. Food Biosci..

[87] Khan A.A., Cui F.-J., Ullah S., Ali K. (2024). Food production and food-related CO2 emissions in China: An examination of the role of economic governance insights from innovative fourier approach. Environment Development and Sustainability.

[88] Khan A.A., Yao F.-J., Cui F.-J., Li Y., Lu L., Khan I., Jalal A., Fang M., Alabbosh K.F., Awad M.F. (2024). Comparative analysis of physicochemical properties and biological activities of crude polysaccharides isolated from selected *Auricularia cornea* strains. Food Biosci..

[89] Zhou X., Zhou X., Zhou L., Jia M., Xiong Y. (2024). Nanofillers in novel food packaging systems and their toxicity issues. Foods.

[90] Juikar S.K., Warkar S.G. (2023). Biopolymers for packaging applications: An overview. Packag. Technol. Sci..

[91] Chatterjee N., Nandi S.K., Dhar P., Ullah A., Ahmed S. (2025). The future of green biopolymers in packaging applications. Green Biopolymers for Packaging Applications.

